# MRI functional and tissue characterisation in patients with systemic lupus erythematosus

**DOI:** 10.1186/1532-429X-14-S1-P196

**Published:** 2012-02-01

**Authors:** Sebastian Buss, Angela Gruber, Hugo A Katus, Dirk Lossnitzer

**Affiliations:** 1 Department of Cardiology, University of Heidelberg, Heidelberg, Germany

## Background

The high prevalence of cardiovascular abnormalities in patients (pts.) with systemic lupus erythematosus (SLE) results in an increased risk of premature cardiovascular events. Inflammatory and immunological processes have been associated with the pathogenesis of myocardial necrosis and dysfunction, however only scarce data exists on cardiac tissue characterization. Non-invasive gadolinium contrast-enhanced cardiovascular MRI (CE-MRI) offers the ability to identify micro-vascular non-infarct-specific inflammatory processes in combination with myocardial functional assessment.

We sought to investigate the utility of cardiac MRI (CMR) for functional and morphological tissue characterisation in SLE pts.

## Methods

We studied 29 SLE pts. (26 females, 44+/-13 years). Classification of pts. followed the criteria of the American College of Rheumatology and assessment of SLE disease was based on the Systemic Lupus Disease Activity Index. CMR was performed on a 1.5T scanner (Philips, Achieva) and included left/right ventricular (LV/RV) functional cine imaging (EF, EDV, ESV) and CE-MRI 10 min. after gadolinium-DTPA injection (0.2mmol/kg bw) using standard MRI protocols. In a 17-segment-model, infarct-typical CE-MRI areas were classified as sub-endocardial lesions, infarct-atypical forms as intramural, patchy, epi-myocardial or pericardial. Functional values were compared with the age-matched Heidelberg normal collective (120 pts.).

## Results

All pts. could be scanned successfully. SLE LV-function and -size (EF, EDV, ESV) were similar when compared to controls (64±9vs.66±6%;153±31vs.151±35ml; 56±24vs.52±18ml; p=n.s.). In contrast, RV-size (EDV,ESV) was significantly higher (178±30vs.155±48ml;84±21vs.67±25ml; p=0.01), albeit with preserved function (53±9vs.57±9%EF). 76% of pts showed LV myocardial CE-MRI (17pts.=infarct-atypical, 3pts.=both forms, 2 pts.=infarct-typical). 11pts. showed pronounced pericardial CE-MRI with concomitant effusions and adhesions.

## Conclusions

The combined functional and morphological MRI approach in SLE pts. identified a considerable amount of myocardial signal abnormalities, in the absence of clinical signs suggesting myocardial involvement. Functionally, although CE-MRI revealed pronounced LV-involvement, LV function and dimensions were preserved. However, increased RV volumes could be detected leading to the hypothesis that other mechanisms such as increased pulmonary hypertension or vascular stiffness have to be taken into account for RV remodelling. Taking into consideration the significant cardiovascular morbidity and mortality in SLE pts., and the difficulty in the early detection of cardiac manifestations, MRI could serve as a non-invasive screening tool to assess cardiac SLE involvement and hence influence disease management and prognosis.

## Funding

None.

